# Identification and validation of biomarkers of Shenggu Zaizao Wan in the treatment of steroid-induced osteonecrosis of the femoral head by integrating network pharmacology and bulk transcriptomic

**DOI:** 10.3389/fmed.2026.1732825

**Published:** 2026-02-04

**Authors:** Tao Ma, Duoxian Wang, Qingsheng Xie, Yin Li, Jinpeng Wang, Xiaogang Zhang, Lingwei Yuan, Hairong He, Xianfu Han, Xuerui Liu, Jianjun Liu, Haiyang Yu, Jinqiu Wu

**Affiliations:** 1 Clinical College of Traditional Chinese Medicine, Gansu University of Chinese Medicine, Lanzhou, China; 2 Department of Sports Medicine, Gansu Provincial Hospital of Traditional Chinese Medicine, Lanzhou, China; 3 Department of Orthopaedics, Affiliated Hospital of Gansu University of Chinese Medicine, Lanzhou, China; 4 Department of Orthopaedics, Shenzhen Bao'an Traditional Chinese Medicine Hospital, Shenzhen, China

**Keywords:** biomarkers, molecular docking, network pharmacology, Shenggu Zaizao Wan, steroid-induced osteonecrosis of the femoral head

## Abstract

**Background:**

Steroid-induced osteonecrosis of the femoral head (SONFH) is a debilitating condition. Research has shown that Shenggu Zaizao Wan (SZW) may have therapeutic effects on SONFH. This study aimed to elucidate the mechanisms by which SZW treats SONFH.

**Methods:**

SONFH and control samples were retrieved from a database to identify differentially expressed genes (DEGs). Biomarkers were obtained through the intersection of DEGs and potential targets, machine learning, and expression validation. A nomogram was constructed, followed by gene set enrichment analysis (GSEA) of biomarkers and immune infiltration analysis. Molecular docking was conducted, and the expression of biomarkers was evaluated using real-time quantitative reverse transcription PCR (RT-qPCR).

**Results:**

A total of 69 potential SZW targets and 1,671 DEGs were identified. IKBKB and PRKCA were established as biomarkers for SONFH, and a nomogram was developed. GSEA indicated that IKBKB and PRKCA may influence pathways related to the ribosome. Immune infiltration analysis revealed that CD4^+^ T cells play a role in SONFH. Molecular docking showed a strong affinity between SZW compounds and biomarkers. The expression of biomarkers in clinical samples aligned with the results from bioinformatics analysis.

**Conclusion:**

This study identified IKBKB and PRKCA as potential biomarkers and targets for SZW treatment in SONFH.

## 1 Introduction

Steroid-induced osteonecrosis of the femoral head (SONFH) is a skeletal disorder caused by prolonged or excessive glucocorticoid use, characterized by the progressive necrosis of femoral head cells. This can ultimately lead to degenerative arthritis and collapse of the femoral head (1). Each year, approximately 20,000 to 30,000 new cases of SONFH are reported globally (2). The disease progresses rapidly and is associated with a high disability rate, resulting in a substantial economic burden on patients and potentially diminishing their long-term quality of life (3). Due to its destructive nature, early intervention in SONFH is crucial. Current treatment strategies primarily involve conservative therapies such as anticoagulants, vasodilators, statins, bisphosphonates, and physical therapy, along with surgical options like core decompression and total hip replacement (4). Although total hip replacement offers a relatively effective solution for advanced cases, postoperative complications and the need for joint revisions exacerbate the patient’s burden, placing additional strain on social healthcare systems (5). Therefore, there is an urgent need for novel therapeutic strategies for SONFH.

In China, traditional Chinese medicine (TCM) has a long history of treating SONFH and plays a significant role in preventing bone cell death and structural damage to the femoral head caused by the condition (6). Shenggu Zaizao Wan (SZW), composed of Epimedium brevicornu Maxim, Davallia trichomanoides Blume, Astragalus membranaceus, Cinnamomi ramulus, Wolfiporia cocos, Rhizoma alismatis, *Salvia miltiorrhiza*, Panax notoginseng, *Crataegus pinnatifida* Bunge, and *Psoralea corylifolia* L., has shown significant clinical efficacy in treating the early and intermediate stages of SONFH. Recent studies suggest that the active components of these herbs are widely used for treating fractures, joint diseases, and various chronic orthopedic conditions, particularly exhibiting notable effects on SONFH (7, 8). Icariin, the active compound in Epimedium, exerts therapeutic effects on femoral head necrosis through anti-inflammatory actions, reduction of vascular damage, prevention of osteocyte apoptosis, promotion of bone marrow mesenchymal stem cell proliferation, reduction of oxidative stress, and inhibition of adipocyte differentiation (9, 10). Additionally, astragaloside IV can alleviate the progression of SONFH by inhibiting endoplasmic reticulum stress (11). Active ingredients such as tanshinone I (TsI) and tanshinone IIA have shown positive effects in antioxidation and antithrombosis (12, 13). Other components also provide vascular protective effects and promote bone repair (14, 15). In recent years, network pharmacology has achieved significant progress in exploring the therapeutic mechanisms of TCM formulas, identifying effective components, and uncovering therapeutic targets. Compared to previous studies on single components, research on the interactions between multiple components and targets offers a more accurate reflection of the *in situ* conditions in clinical practice (16). Despite these advancements, the treatment of SONFH with SZW remains based on TCM theory (17) and lacks modern pharmacological research, leaving its molecular mechanisms unclear. Identifying new targets and molecular mechanisms of SZW and its active components in the treatment of SONFH is of significant importance.

In this study, transcriptomic data related to SONFH were obtained from the Gene Expression Omnibus (GEO) database, and differentially expressed genes (DEGs) were identified. Disease targets associated with SONFH and targets of SZW acting on SONFH were sourced from the Traditional Chinese Medicine Systems Pharmacology Database and Analysis Platform (TCMSP), Herbal Encyclopedia for Research on Bioactivity and Chemical Constituents (HERB), and GeneCards databases. These targets were then intersected with DEGs to identify candidate genes. Machine learning and expression validation methods were employed to screen potential biomarkers. By integrating the results of Kyoto Encyclopedia of Genes and Genomes (KEGG) enrichment analysis and the protein–protein interaction (PPI) network, the potential molecular mechanisms of these biomarkers in SONFH were elucidated. Finally, molecular docking was used to evaluate the binding affinity of biomarkers to the active ingredients in SZW, verifying the molecular mechanisms of SZW and its active components in the treatment of SONFH. The findings have significant theoretical and practical implications for improving clinical treatments and developing innovative targeted therapeutic agents.

## 2 Materials and methods

### 2.1 Data collection

The GEO database^1^ provided transcriptome data for SONFH (GSE123568 and GSE74089). The GSE123568 dataset (GPL15207) included 30 SONFH and 10 control blood samples, serving as the training set, while the GSE74089 dataset (GPL13497) contained 4 ONFH and 4 control cartilage tissue samples, used as the validation set.

### 2.2 Sifting of targets and active components of SZW

SZW consists of 10 components: *Epimedium brevicornu Maxim, Davallia trichomanoides Blume, Astragalus membranaceus*, *Cinnamomi ramulus, Wolfiporia cocos, Rhizoma alismatis, Salvia miltiorrhiza, Panax notoginseng, Crataegus pinnatifida Bunge,* and *Psoralea corylifolia L*. Active components and targets for *Epimedium brevicornu Maxim, Davallia trichomanoides Blume, Astragalus membranaceus*, *Cinnamomi ramulus, Wolfiporia cocos, Rhizoma alismatis, Salvia miltiorrhiza,* and *Panax notoginseng* were obtained from the TCMSP database^2^, using criteria for oral bioavailability (OB) ≥ 30% and drug likeness (DL) ≥ 0.18. Therapeutic targets for *Crataegus pinnatifida Bunge* and *Psoralea corylifolia L*. were retrieved from the HERB database.^3^ After integrating all drug targets, standardized SZW targets were acquired *via* the UniProt database.^4^ A drug-component-target network was constructed using Cytoscape (v 3.10.2) (18) with topological data sourced from TCMSP.

### 2.3 Screening putative targets of SZW for SONFH intervention

The Genecards database^5^ was used to obtain SONFH-related targets by merging and deduplicating data with keywords “steroid-induced osteonecrosis of the femoral head” and “femur head steroid necrosis.” The final SONFH targets were obtained after merging and deduplication. SZW therapeutic targets for SONFH were identified by intersecting disease-associated targets with compound targets using the VennDiagram package (v 1.7.3) (19). A drug-target-disease network was established using Cytoscape (v 3.10.2) (18).

### 2.4 Identification of DEGs and candidate genes

The “limma” package (v 3.54.0) (20) was used to identify differentially expressed genes (DEGs) between SONFH and control samples (SONFH vs. control) (|log_2_FoldChange (FC)| > 0.5, *p* < 0.05) from all samples in the GSE123568 dataset. Volcano plots were generated using ggplot2 (v3.3.6) (21) to visualize DEGs, with the ten most significantly up/down-regulated genes annotated. Hierarchical clustering of the top 10 up−/down-regulated genes in SONFH vs. controls was visualized using the “ComplexHeatmap” package (v 2.14.0) (22). Candidate genes were identified by intersecting SONFH-related DEGs with SZW therapeutic targets using “VennDiagram” (v 1.7.3).

### 2.5 Enrichment analysis and construction of PPI network

Candidate genes underwent functional annotation and pathway enrichment analysis using KEGG and GO databases *via* the “clusterProfiler” package (v 4.6.2) (23), with statistical significance set at *p* < 0.05. The top 5 enriched terms in GO and the top 10 terms in KEGG were visualized. A protein–protein interaction (PPI) network was constructed using the Search Tool for the Retrieval of Interacting Genes and Proteins (STRING) database^6^ (confidence ≥0.4) to examine the protein-level interactions of candidate genes. The analysis was restricted to *Homo sapiens*, and visual outputs were generated using Cytoscape v3.10.2.

### 2.6 Machine learning and expression level verification

Biomarker discovery integrated the LASSO regression algorithm, SVM-RFE algorithm, and transcriptomic profiling. LASSO regression (*via* glmnet v4.1-4) (24) and SVM-RFE (svmRadial method using caret v6.0-93) (25) were applied to the GSE123568 cohort. Candidate biomarkers were identified by intersecting genes selected by both algorithms using the “VennDiagram” package (v 1.7.3). Gene expression differences between SONFH and control samples in both the GSE123568 and GSE74089 cohorts were assessed using Wilcoxon testing (*p* < 0.05). Visualization of the results was performed using the “ggplot2” package (v 3.3.6). Genes showing significant differences and consistent expression trends across both datasets were considered biomarkers. For chromosomal localization, RCircos (v 1.2.2) (26) generated genome-wide distribution plots.

### 2.7 Construction of the nomogram model

Using the “rms” package (v 6.5-0) (27), a biomarker-driven nomogram was developed to assess diagnostic performance in the GSE123568 dataset. Each biomarker was assigned a score, and the sum of the scores represented the total points. The likelihood of SONFH was predicted based on the total points. The accuracy of the nomogram was evaluated using a calibration curve (*via* the “ResourceSelection” package v 0.3.5)^7^ and the Hosmer-Lemeshow (HL) test (*p* > 0.05, mean absolute error [MAE] < 0.1). Additionally, ROC analysis using pROC v1.18.0 (28) evaluated the diagnostic efficacy of the nomogram (Area Under Curve [AUC] > 0.7).

### 2.8 GSEA

Gene set enrichment analysis (GSEA) was employed to explore the functional roles of biomarkers in SONFH. The reference gene set used was “c2.kegg.v7.4.symbols,” obtained from the Molecular Signatures Database (MSigDB, https://www.gsea-msigdb.org/gsea/msigdb/). Spearman correlation coefficients (using the psych v2.4.3 package) (28) ranked genes based on biomarker association strength. GSEA (*via* clusterProfiler v4.6.2) on GSE123568 identified significant pathways (P/FDR < 0.05), with the top 10 entries prioritized by *p*-value.

### 2.9 Analysis of immune cell type abundance

Immune cell infiltration was assessed by ssGSEA for 28 immune cell types (29) in all SONFH and control samples from the GSE123568 dataset. Differential immune cells (DICs) were identified *via* the Wilcoxon test (*p* < 0.05). Correlations between biomarkers and DICs, as well as correlations among DICs, were evaluated using Spearman correlation analysis (*via* the “psych” package v 2.2.9) (30) (|correlation coefficient (cor)| > 0.3, *p* < 0.05). The results were visualized using the “ggplot2” package (v 3.3.6).

### 2.10 Prediction of factors linked to biomarkers

Transcription factors (TFs) linked to biomarkers were obtained from the NetworkAnalyst database.^8^ miRNAs were retrieved from the miRWalk database^9^ and miRTarBase^10^, with miRNAs linked to biomarkers identified by overlapping results from both databases. The lncRNAs associated with these miRNAs were obtained from the miRNet database.^11^ All data were visualized as previously described.

### 2.11 Molecular docking

Molecular docking simulations were conducted to assess the binding affinities between prioritized bioactive compounds from SZW and candidate biomarkers. 3D structural coordinates of target biomarkers were retrieved from the RCSB PDB repository^12^, while the three-dimensional conformations of bioactive compounds were sourced from PubChem.^13^ Docking simulations were performed using the CB-DOCK platform^14^, which automatically executed the entire process, including protein preparation (removal of water molecules and addition of hydrogen atoms), active pocket prediction, grid generation, conformational searching, and scoring. Binding interactions between the bioactive ligands and target biomarkers were analyzed, with a total score threshold of <−5.0 (31).

### 2.12 RT-qPCR analysis

Biomarker expression was evaluated in blood samples using RT-qPCR. Five SONFH and five control blood samples were collected at the Gansu University of Traditional Chinese Medicine Affiliated Hospital. Informed consent was obtained from all participants, and ethical approval was granted by the Ethics Committee of Gansu University of Traditional Chinese Medicine Affiliated Hospital in May 2023 (protocol code “202,345”). Total RNA was extracted from the paired samples using TRIzol reagent (Vazyme, Nanjing, China), and RNA concentrations were measured by Yi Sheng, Wuhan, China. Complementary DNA (cDNA) was synthesized from mRNA templates through reverse transcription. The cDNA was then diluted with ddH_2_O and subjected to RT-qPCR using the following program: pre-denaturation at 95 °C for 1 min, followed by 40 cycles of denaturation at 95 °C for 20 s, annealing at 55 °C for 20 s, and extension at 72 °C for 30 s. The expression levels of biomarkers in SONFH versus control samples were calculated using the 2^−ΔΔCt^ method, and expression differences were assessed using Student’s *t*-test (*p* < 0.05). All data processing and visualization were performed using GraphPad Prism software (version 8.0) (32). Detailed primer information and machine testing conditions are provided in Supplementary Table 1.

### 2.13 Statistical analysis

All statistical analyses and data visualizations were performed using R software (v4.2.2). Specifically, differential expression analysis was conducted using the “limma” package (v 3.54.0); feature selection *via* LASSO regression was performed using the “glmnet” package (v4.1-4), while support vector machine-recursive feature elimination (SVM-RFE) was conducted with the “caret” package (v6.0-93); functional and pathway enrichment analyses were carried out using the “clusterProfiler” package (v 4.6.2); intersections were visualized with the “VennDiagram” package (v 1.7.3); a nomogram model based on key biomarkers was constructed using the “rms” package (v 6.5-0); and Spearman correlation analysis was performed using the “psych” package (v 2.2.9). In addition, drug-component-target networks and PPI networks were built and visualized using Cytoscape software (v 3.10.2). For comparisons between groups, Student’s *t*-test or Wilcoxon rank-sum test was applied according to the data distribution, with a *p*-value < 0.05 considered statistically significant.

## 3 Results

### 3.1 Identification of the potential targets of SZW in the treatment of SONFH

**Figure 1:**
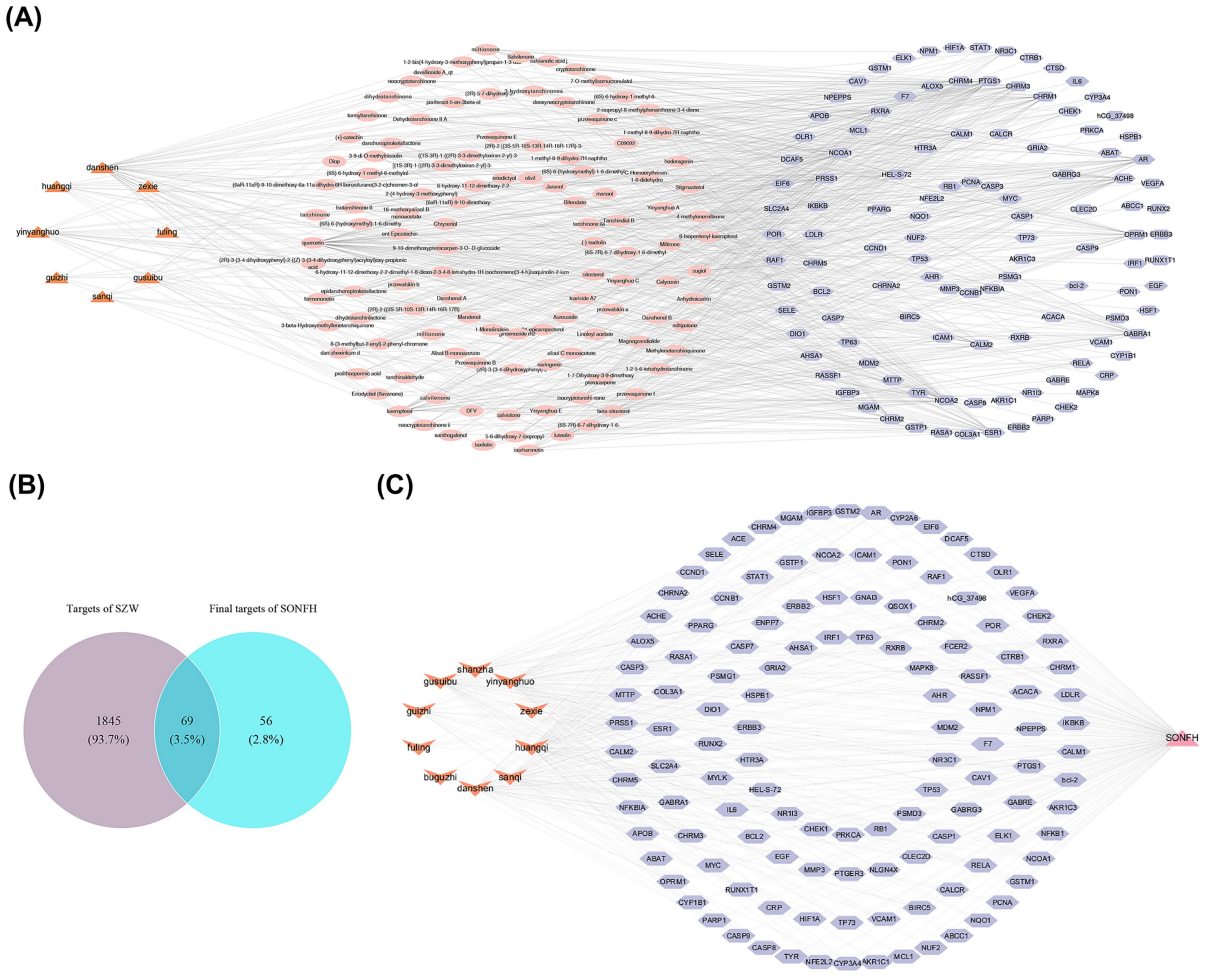
Identification of potential targets of SZW in the treatment of SONFH. **(A)** Drug-active ingredient-target interaction network diagram. Orange represented drugs, pink represented active ingredients, and purple represents targets. **(B)** The Venn diagram of targets of Shenggu Zaizao Wan and SONFH. **(C)** Drug-target-disease interaction network diagram. Orange represented drugs, purple represented targets, and pink represented diseases.

In the TCMSP database, 283 targets and 124 active ingredients were obtained from 8 drugs after deduplication (Supplementary Tables 1, 2). The HERB database provided 13 target proteins. After consolidating and standardizing data from both databases, 125 high-confidence therapeutic targets for SZW were identified (Supplementary Table 3). The network constructed based on the TCMSP database results revealed relationships between 8 drugs, 104 active ingredients (e.g., hederagenin), and 115 targets (e.g., ACACA). In this network, red, purple, and pink represented the 8 drugs, 115 targets, and 104 active ingredients, respectively (Figure 1A). Subsequently, 1,914 disease-related targets were sourced from the GeneCards database (Supplementary Table 4). After intersecting these disease-related targets with compound targets, 69 potential targets were identified as implicated in SZW treatment for SONFH (Figure 1B). The relationships among the drugs, disease, and target genes are shown in Figure 1C, where orange, purple, and pink represent drugs, targets, and diseases, respectively.

### 3.2 Screening and preliminary analysis of candidate genes

**Figure 2:**
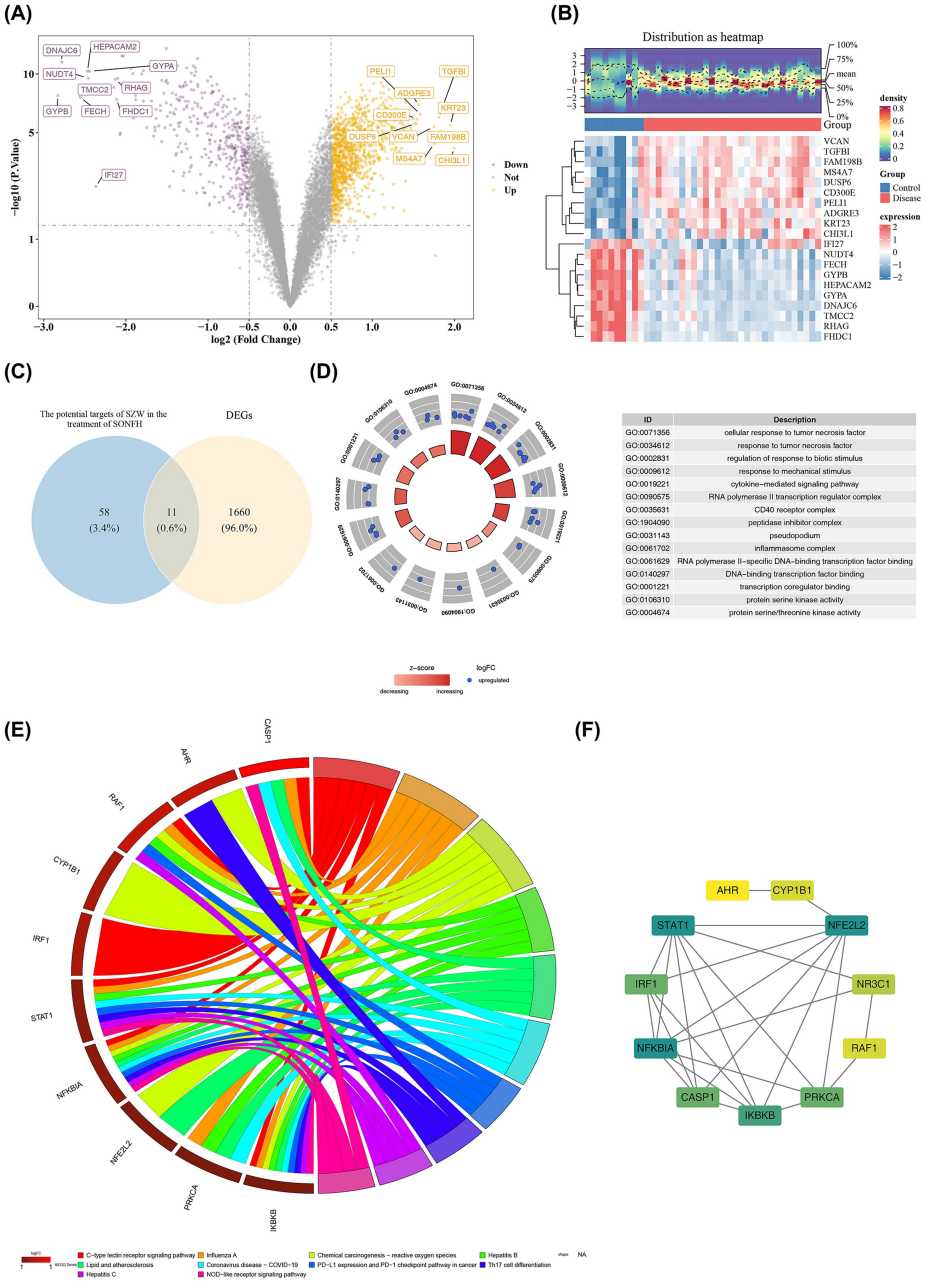
Screening and preliminary analysis of candidate genes. **(A)** Volcanic map of differential gene expression between SONFH group and control group. Yellow: Up-regulated gene; Purple: Down-regulated gene. The names of the top 10 genes up and down regulated were displayed. **(B)** Differential gene expression heatmap between SONFH group and control group. The color represented the expression level of the gene, with higher expression levels indicating a redder color and lower expression levels indicating a bluer color. Red: Disease group; Blue: Control group. **(C)** Potential targets of Shenggu Zaizao Wan in the treatment of SONFH and the Venn diagram of DEGs. **(D)** Gene GO enrichment circle diagram of candidate genes. Each GO entry had a corresponding circle, and the color inside the circle represented the logFC value of the entry, where blue represented upregulation expression. **(E)** KEGG pathway enrichment string plot of candidate genes. Each line represented a different molecular mechanism. **(F)** PPI network diagram. The degree of the protein encoded by genes corresponded to colors ranging from yellow to green from small to large.

The GSE123568 dataset revealed 1,671 DEGs (|log_2_FC| > 0.5, *p* < 0.05), comprising 1,245 up-regulated and 426 down-regulated genes in SONFH samples (Figures 2A,B). Intersection with SZW therapeutic targets identified 11 core candidate genes (Figure 2C). Gene Ontology (GO) analysis highlighted 499 biological processes (BPs), 5 cellular components (CCs), and 56 molecular functions (MFs) significantly enriched (*p* < 0.05) (Figure 2D; Supplementary Table 5). For example, candidate genes were enriched in biological processes related to cytokine-mediated signaling, cellular components such as the CD40 receptor complex, and molecular functions like protein serine/threonine kinase activity. In KEGG analysis, candidate genes were notably enriched in the C-type lectin receptor signaling pathway (*p* < 0.05) (Figure 2E; Supplementary Table 6). The PPI network indicated that the 11 genes were interconnected, with NFE2L2, NFKBIA, and IKBKB showing interactions with multiple genes (Figure 2F).

### 3.3 Identification of biomarkers

**Figure 3:**
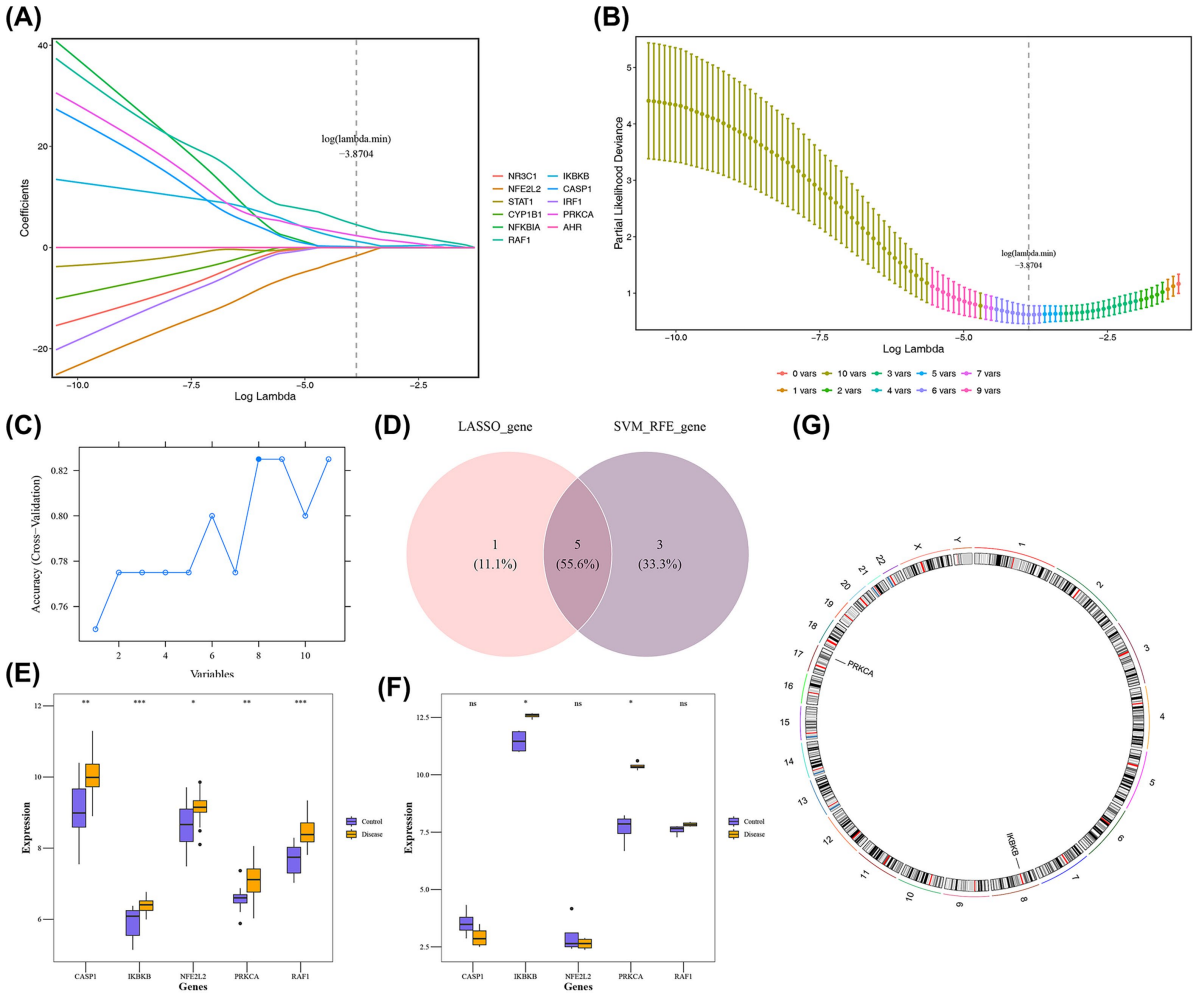
Identification of biomarkers **(A,B)** Lasso analysis of candidate genes. When reaching the optimal lambda, remove variables with coefficients equal to 0. **(C)** Construction of SVM-RFE model. The blue solid dots represented the potential feature genes screened out when the model reached the highest accuracy after the first 8 features. **(D)** Venn diagram of characteristic genes of LASSO and SVM-RFE. **(E,F)** Box plot of expression levels of potential key genes in the training set and validation set. Purple: Control group; Yellow: Disease group. ns: *p* ≥ 0.05; *: *p* < 0.05; **: *p* < 0.01; ***: *p* < 0.001. **(G)** Chromosome localization analysis of key genes. The outer circle of the image corresponded to the position of the chromosome, while the inner circle represented the position information of key genes on the chromosome.

Feature selection through least absolute shrinkage and selection operator (LASSO) (log(lambda.min) = −3.8704) identified 6 key genes (Figures 3A,B), complemented by SVM-RFE revealing 8 biomarkers (Figure 3C). A total of 5 candidate biomarkers (NFE2L2, RAF1, IKBKB, CASP1, and PRKCA) were selected from the intersection of the two algorithms (Figure 3D). Notably, IKBKB and PRKCA showed significant expression differences between SONFH and control samples (*p* < 0.05), with both genes being upregulated in SONFH across two independent datasets (Figures 3E,F). Therefore, IKBKB and PRKCA were designated as biomarkers. Chromosomal location analysis revealed that IKBKB is located on chromosome 8, while PRKCA is located on chromosome 17 (Figure 3G).

### 3.4 Construction of a SONFH nomogram

**Figure 4:**
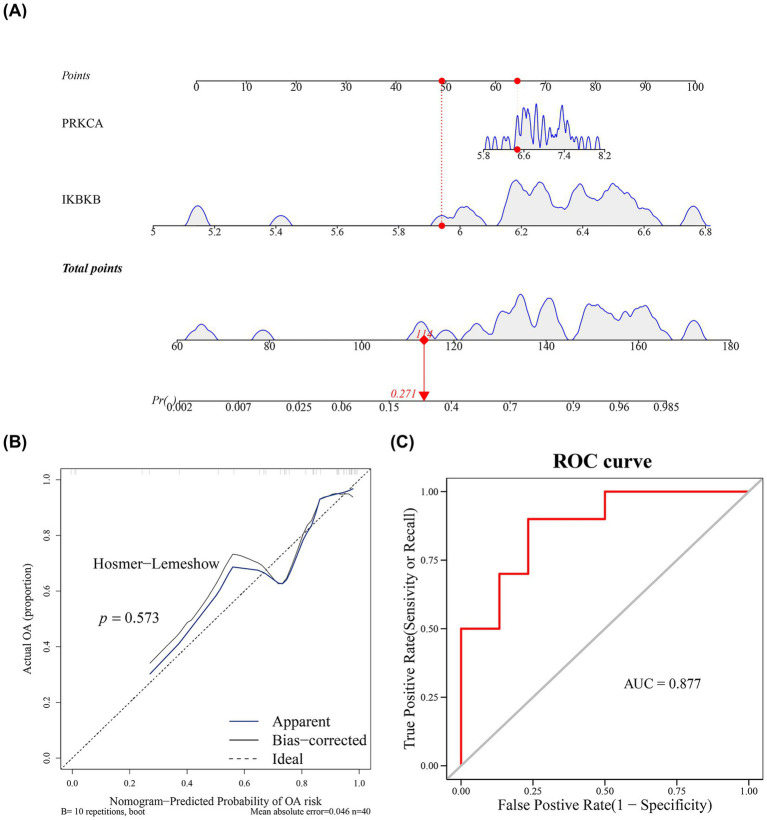
Construction of nomogram. **(A)** Nomogram of key genes. The horizontal axis of two genes represented the expression level of the genes. **(B)** Calibration curve of key genes. The black dashed line represented a perfect prediction. The blue solid line represented the entire queue, and the black solid line is used for deviation correction through bootstrap (1,000 repetitions), indicating the observed column chart performance. **(C)** ROC curve analysis chart. The closer the AUC value was to 1, the stronger the predictive performance.

Based on the biomarkers IKBKB and PRKCA, a diagnostic nomogram model was constructed to assess SONFH risk (Figure 4A). The calibration curve demonstrated good fit (*p* = 0.573) and low error (MAE = 0.046) (Figure 4B). ROC analysis using the GSE123568 dataset, which was employed to build the model, showed that the model exhibited strong discriminative ability (AUC = 0.877) (Figure 4C). However, the performance evaluation of this model is based solely on internal data at this stage, and its diagnostic efficacy and clinical applicability require further validation.

### 3.5 Enrichment pathways of biomarkers

**Figure 5:**
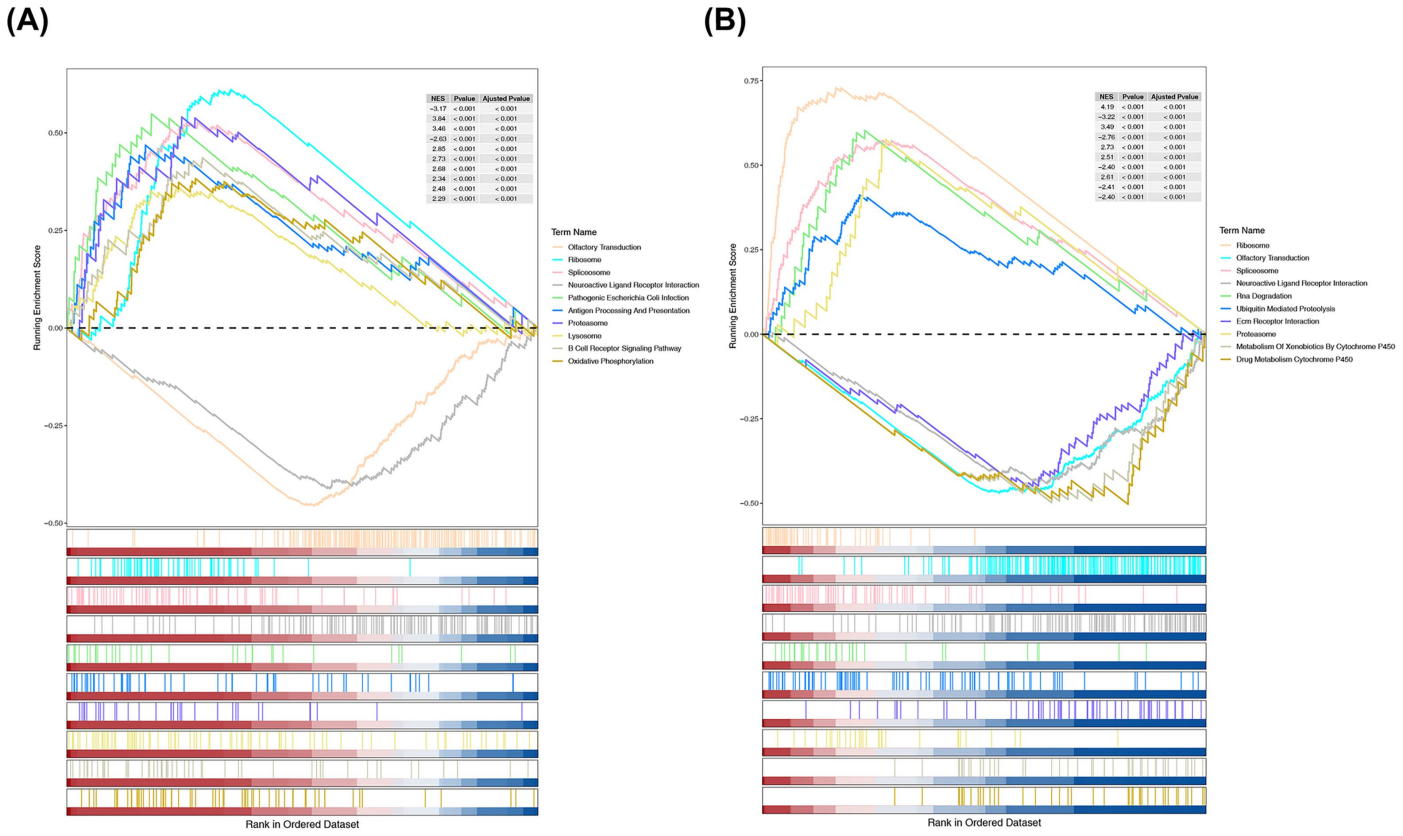
GSEA enrichment analysis of IKBKB **(A)** and PRKCA **(B)**. Part 1: the top 5 or 2 lines are the line chart of gene enrichment score. The vertical axis represented the corresponding running ES, and there was a peak in the line graph, which was the enrichment score of this gene set. The genes before the peak were the core genes under this gene set. The horizontal axis represented each gene in this gene set, corresponding to the vertical line resembling a barcode in the second part; Part 2: the barcode like part is hits, with each vertical line corresponding to a gene in the gene set.

GSEA revealed that IKBKB and PRKCA were significantly enriched in 62 and 53 pathways, respectively (Supplementary Table 7, false discovery rate [FDR] < 0.05, *p* < 0.05). Specifically, IKBKB was enriched in oxidative phosphorylation (Figure 5A), while PRKCA was enriched in RNA degradation and ubiquitin-mediated proteolysis (Figure 5B). Additionally, both IKBKB and PRKCA were enriched in pathways related to the ribosome, spliceosome, and neuroactive ligand receptor interactions. These results suggest that IKBKB and PRKCA may play a significant role in the progression of SONFH through these enriched pathways.

### 3.6 Immune cells linked to biomarkers in SONFH

**Figure 6:**
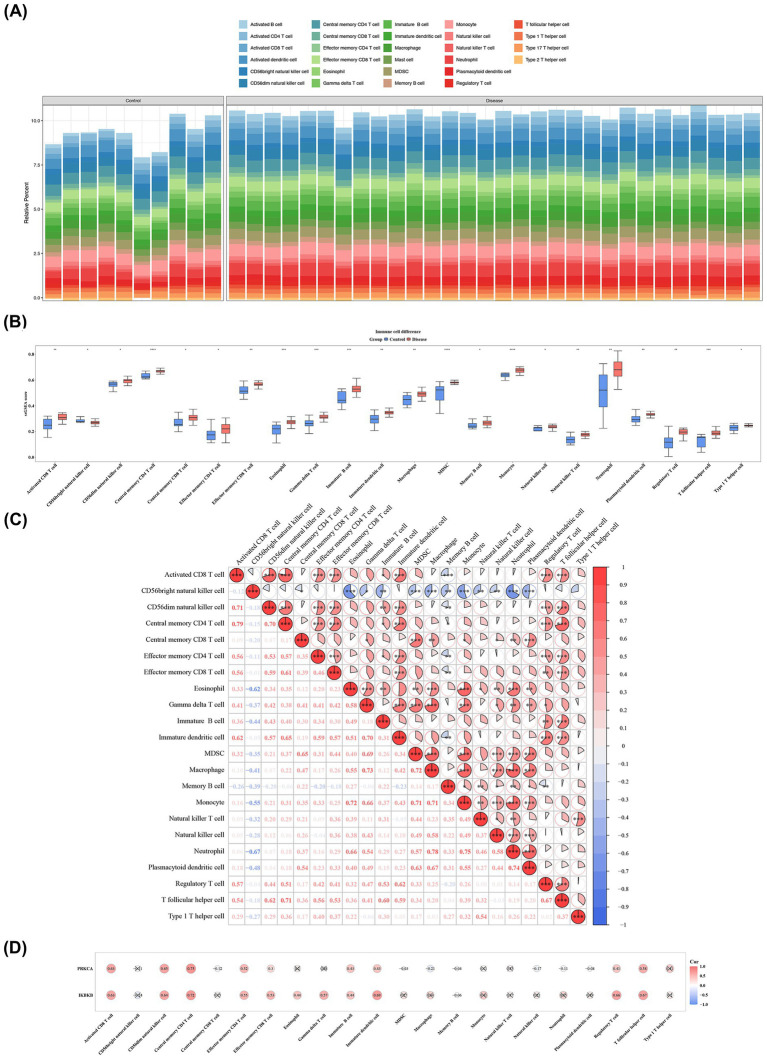
Immune cells linked to biomarkers in SONFH. **(A)** The proportion of immune cells in each sample. **(B)** Twenty-two immune infiltrating cells based on enrichment score boxplots between disease group and control group. Red: Disease group; Blue: Control group. *: *p* < 0.05 **: *p* < 0.01; ***: *p* < 0.001; ****: *p* < 0.0001. **(C)** Correlation heatmap between 22 different immune cells. The redder the color, the stronger the positive correlation, and the bluer the color, the greater the negative correlation coefficient. *: *p* < 0.05; **: *p* < 0.01; ***: *p* < 0.001. **(D)** Correlation heatmap between 22 differential immune cells and 2 biomarkers. The redder the color, the stronger the positive correlation, and the bluer the color, the greater the negative correlation coefficient.

To evaluate the systemic immune status of patients with SONFH, the relative abundance of 28 immune cell subsets was quantified using single-sample GSEA (ssGSEA) based on whole-blood transcriptome data from the GSE123568 dataset. As illustrated in Figure 6A, distinct patterns were observed in the abundance profiles of various immune cell subtypes between the SONFH and control groups. Wilcoxon analysis revealed significant differences in the abundance of 22 immune cell subsets (e.g., activated CD8^+^ T cells) between SONFH and control groups (*p* < 0.05) (Figure 6B). Correlation analysis indicated a significant positive association between activated CD8^+^ T cells and central memory CD4^+^ T cells (cor = 0.79, *p* < 0.001), as well as between macrophages and neutrophils (cor = 0.78, *p* < 0.001) (Figure 6C; Supplementary Table 8). Further analysis showed robust positive correlations between IKBKB (cor = 0.72, *p* < 0.01) and PRKCA (cor = 0.75, *p* < 0.01) with central memory CD4^+^ T cells (Figure 6D; Supplementary Table 9). These results suggest that these differentially infiltrated cells (DICs), particularly central memory CD4^+^ T cells, may have a significant impact on SONFH.

### 3.7 Construction of network

**Figure 7:**
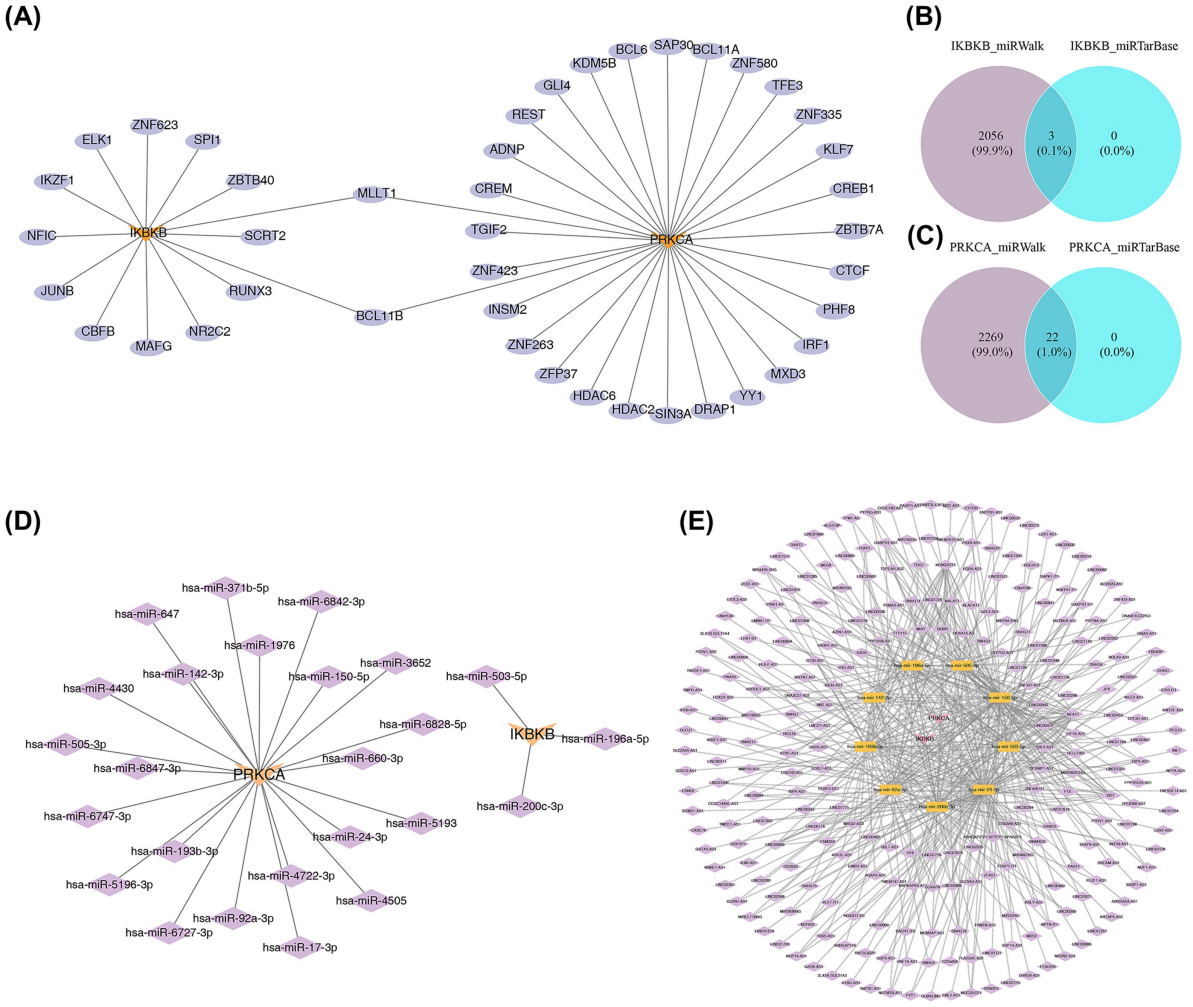
Construction of network. **(A)** TF-mRNA regulatory network. Orange represented mRNA, purple represented predicted TF. **(B,C)** Venn diagram of key miRNAs. **(D)** miRNA-key gene relationship network. Orange represented key genes, while purple represented predicted miRNAs. **(E)** lncRNA-miRNA-mRNA interaction network diagram. Orange represented miRNA, purple represented predicted lncRNA, and pink represented predicted lncH19 of particular interest.

A total of 14 TFs, including SPI1 and SCRT2, were linked to IKBKB, while 30 TFs, such as ADNP, were associated with PRKCA (Figure 7A). Among these, two TFs, MLLT1 and BCL11B, were predicted to be linked to FBP1 and RHOG. Furthermore, three miRNAs, including hsa-miR-196a-5P, were found to be associated with IKBKB (Figures 7B,D). A total of 22 miRNAs, such as hsa-miR-24-3p and hsa-miR-17-3p, were linked to PRKCA (Figures 7C,E). Additionally, nine miRNAs were found to have corresponding lncRNAs, with 260 lncRNAs (including MDS2, H19, and SNHG11) linked to these miRNAs. In total, the biomarkers-miRNAs-lncRNAs network incorporated 9 miRNAs, 260 lncRNAs, and the two biomarkers (Figure 7). These factors associated with the biomarkers may play important roles in the progression of SONFH.

### 3.8 Molecular docking between active components of SZW with biomarkers

**Figure 8:**
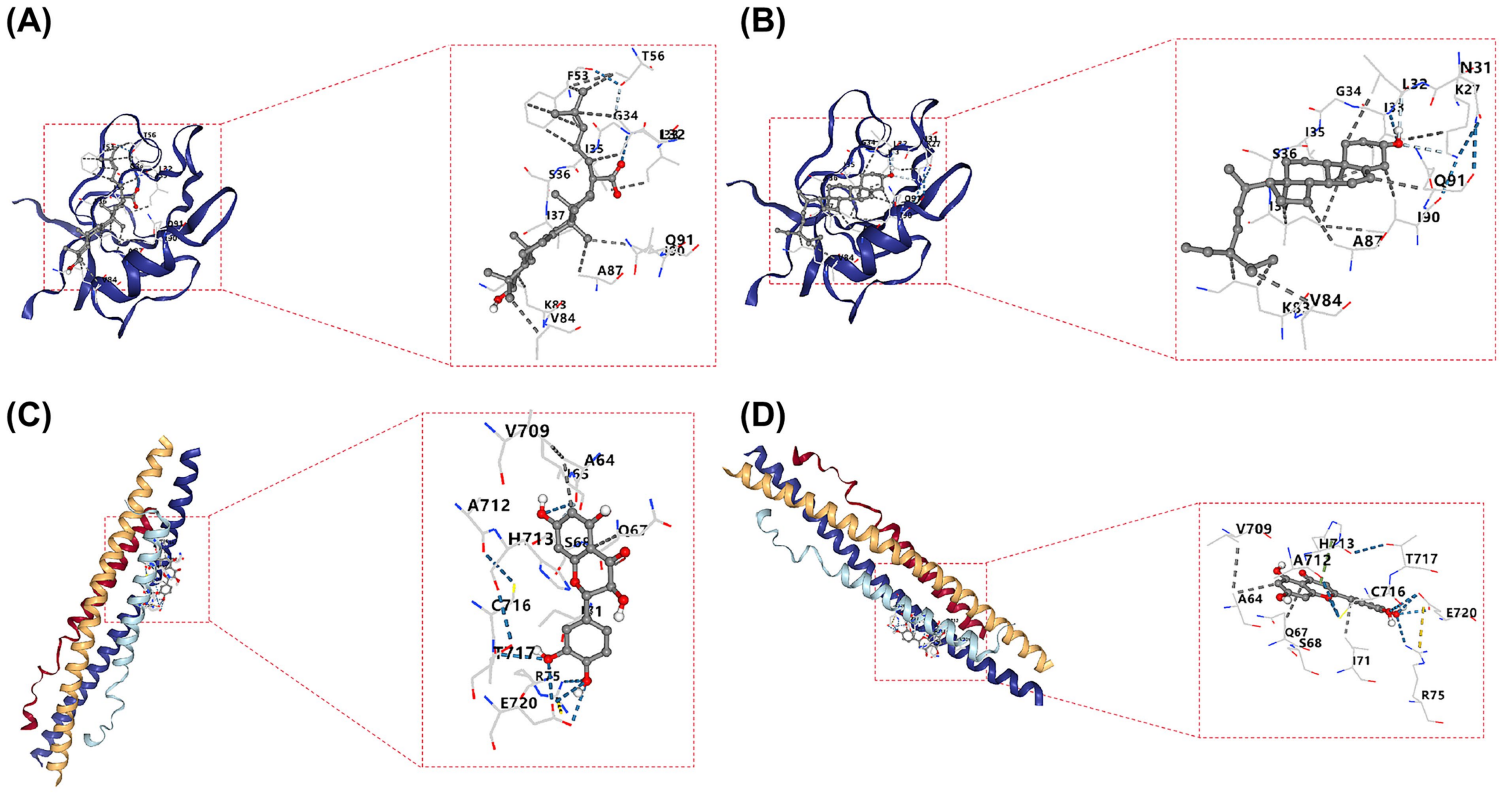
Molecular docking between active components of SZW with biomarkers. **(A)** Molecular docking of trametenolic acid with PRKCA. **(B)** Molecular docking of tanshinone IIA with PRKCA. **(C)** Molecular docking of (-)-taxifolin with IKBKB. **(D)** Molecular docking of luteolin with IKBKB. The number represented the target when combined. The dashed line represented the binding site.

A total of 14 active ingredients of SZW were selected for molecular docking with IKBKB and PRKCA, and the total scores are presented in Table 1. Trametenolic acid (total score = −7.7) (Figure 8A) and tanshinone IIA (total score = −7.6) (Figure 8B) exhibited stronger binding affinity with PRKCA compared to other active ingredients, while (−)-taxifolin (total score = −7.1) (Figure 8C) and luteolin (total score = −6.9) (Figure 8D) demonstrated greater binding affinity with IKBKB. Additional findings are provided in Supplementary Figure 1. These results suggest that these active ingredients may more effectively modulate the biological activity of SONFH-related targets in the respective drugs.

**Table 1:** Molecular docking score.

Active ingredients of SZW	Drugs	Gene	Total score
24-epicampesterol	Epimedium brevicornu Maxim	IKBKB	−6.6
		PRKCA	−7.1
Pinoresinol	Davallia trichomanoides Blume	IKBKB	−6.3
		PRKCA	−5.9
Mairin	Astragalus membranaceus	IKBKB	−6.4
		PRKCA	−7
(−)-taxifolin	Cinnamomi ramulus	IKBKB	−7.1
		PRKCA	−7
Trametenolic acid	Wolfiporia cocos	IKBKB	−6.8
		PRKCA	−7.7
Sitosterol	Rhizoma alismatis	IKBKB	−6.4
		PRKCA	−7
Poriferasterol	*Salvia miltiorrhiza*	IKBKB	−6.6
		PRKCA	−7.2
DFV	Panax notoginseng	IKBKB	−6.3
		PRKCA	−6.7
Bakuchalcone	*Psoralea corylifolia*	IKBKB	−6.8
		PRKCA	−7.2
Quercetin	Epimedium brevicornu Maxim, Astragalus membranaceus, Panax notoginseng	IKBKB	−6.7
		PRKCA	−6.9
Kaempferol	Epimedium brevicornu Maxim, Davallia trichomanoides Blume, Astragalus membranaceus	IKBKB	−6.3
		PRKCA	−7
Luteolin	Epimedium brevicornu Maxim, Davallia trichomanoides Blume, *Salvia miltiorrhiza*	IKBKB	−6.9
		PRKCA	−6.8
Tanshinone IIA	*Salvia miltiorrhiza*	IKBKB	−6.5
		PRKCA	−7.6

### 3.9 Real-time quantitative reverse transcription PCR (RT-qPCR) experiments of biomarkers

**Figure 9:**
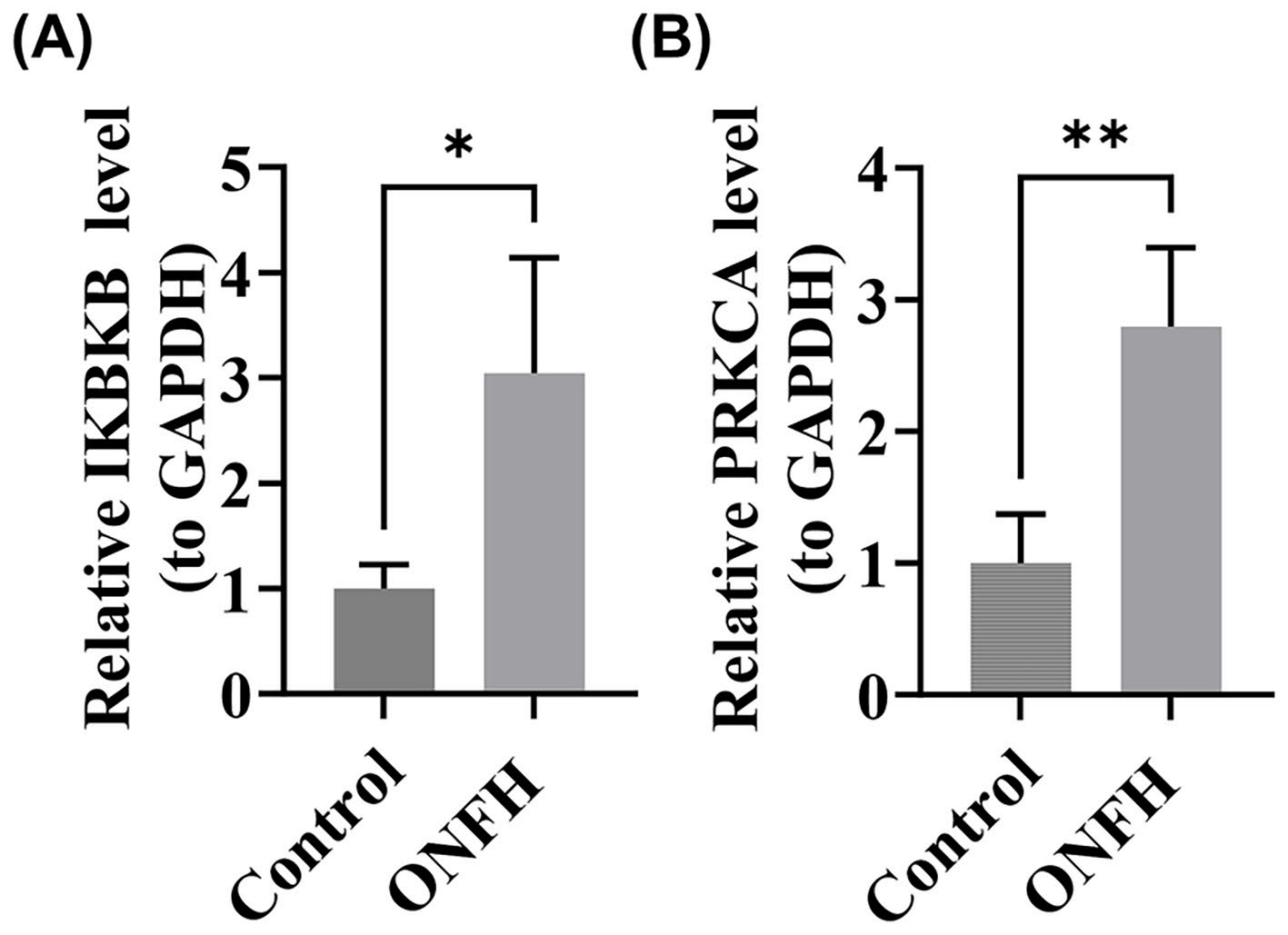
Real-time quantitative reverse transcription PCR (RT-qPCR) experiments of biomarkers. **(A)** IKBKB. **(B)** PRKCA. *: *p* < 0.05, **: *p* < 0.01.

RT-qPCR experiments with clinical samples revealed significant differences in the expression of IKBKB and PRKCA between SONFH and control samples (*p* < 0.05) (Figures 9A,B). The expression patterns of these genes were consistent with the results of our bioinformatics analysis, further validating the reliability of our current findings.

## 4 Discussion

SONFH is a destructive osteoarticular disease caused by long-term and excessive glucocorticoid use, resulting in femoral head collapse, hip pain, and functional impairment (33). The treatment of SONFH presents numerous challenges, highlighting the need for new therapeutic strategies. Using network pharmacology, this study initially identified 283 targets and 124 active components, with Inhibitor of Nuclear Factor Kappa B Kinase Subunit Beta (IKBKB) and Protein Kinase C Alpha (PRKCA) identified as key biomarkers for the therapeutic effects of SZW on SONFH. Additionally, the potential molecular mechanisms of these biomarkers in SONFH were analyzed through KEGG enrichment analysis and PPI network construction. GSEA results suggested that IKBKB and PRKCA may be involved in SONFH progression through pathways related to ribosomes, spliceosomes, and other mechanisms. Immune infiltration analysis revealed that activated CD8^+^ T cells, central memory CD4^+^ T cells, and other immune cell types may significantly impact SONFH. Molecular docking results further demonstrated that certain active ingredients of SZW exhibit strong binding affinities with these biomarkers. Additionally, RT-qPCR analysis showed significant differences in the expression levels of IKBKB and PRKCA between SONFH and control samples. The core innovation of this study lies in the identification of the association between the clinical formula SZW and two key signaling molecules (IKBKB and PRKCA), whose roles in SONFH have not been fully elucidated. This study provides modern scientific evidence for the traditional Chinese herbal compound SZW, laying a theoretical foundation for further mechanistic exploration and optimization of its clinical application.

IKBKB encodes the IKKβ protein, which functions as the central kinase in the NF-κB classical signaling pathway. Along with IKKα and NEMO (IKKγ), IKKβ forms the IKK kinase complex responsible for phosphorylating and degrading the Iκ-Bα protein. This degradation releases RelA/p50 dimers, allowing them to translocate to the nucleus and regulate the expression of target genes (34). Biallelic mutations in IKBKB that result in loss of function cause severe combined immunodeficiency, characterized by reduced T cell populations, developmental abnormalities, and impaired immune responses (35). Additionally, IKBKB plays a pivotal role in B cell maturation, immune responses, cell survival, and the production of antigen-specific antibodies (36). It is also involved in antigen presentation and immune functions in dendritic cells and macrophages, emphasizing its widespread influence on the immune system (37, 38). Previous research has shown that NF-κB signaling mediated by NF-κB-inducing kinase (NIK) is vital for osteoclast differentiation (39). A study further indicated that circIKBKB is upregulated in breast cancer bone metastasis tissues, and its overexpression enhances osteoclastogenesis, promoting the formation of a pre-metastatic niche (40). Moreover, studies on SONFH have demonstrated a significant correlation between IKBKB expression and immune cell function (41). The present study found a notable increase in IKBKB transcription levels in patients with SONFH, and clinical sample validation further corroborated these findings, suggesting that IKBKB plays a role in SONFH progression. Molecular docking analysis in this study revealed that the active components taxifolin and luteolin in SZW have a high binding affinity for the IKBKB protein, suggesting that SZW may regulate IKBKB. Existing literature reports that both taxifolin and luteolin inhibit the activation of the NF-κB signaling pathway (42, 43). Based on the observed upregulation of IKBKB in SONFH and its role in the NF-κB pathway, SZW may target IKBKB through components like taxifolin and luteolin, modulating its kinase activity and preventing excessive NF-κB activation. This could be a potential mechanism through which SZW alleviates inflammation in SONFH, regulates immune cell function, and suppresses abnormal bone resorption. However, this hypothesis requires experimental validation in SONFH models to directly confirm whether SZW and its specific components exert therapeutic effects *via* IKBKB.

PPRKCA is a member of the serine/threonine-specific protein kinase C family, involved in mediating cell growth and inflammatory responses (44). Overexpression of PRKCA inhibits the secretion of lipopolysaccharide (LPS)-induced inflammatory factors, including IL-1β, TNF-*α*, and IL-6, thereby alleviating sepsis-induced acute lung injury in mice. PRKCA and its encoded protein kinase (PKCα) primarily regulate cellular proliferation, differentiation, and anti-apoptotic signaling, making them pivotal in tumor formation and chemotherapy resistance (45). PKCα inhibits osteoblast differentiation while promoting osteoblast proliferation (46), highlighting its role in regulating the cellular signaling involved in osteoblast differentiation and proliferation. However, the detailed molecular regulatory mechanisms of PRKCA remain unclear, requiring further investigation, particularly in the context of osteonecrosis of the femoral head. This study identified a significant upregulation of PRKCA expression in both SONFH clinical samples and public transcriptomic data (GSE123568), suggesting its potential involvement in the pathological progression of SONFH. Similarly, molecular docking results revealed high binding affinity between tanshinone IIA, an active component of SZW, and PRKCA. Previous studies have confirmed that tanshinone IIA inhibits osteoclast differentiation and bone resorption (47). Based on these findings, SZW may modulate PRKCA through components such as tanshinone IIA, influencing its downstream signaling pathways. This mechanism could help regulate the balance between osteogenesis and osteoclastogenesis, potentially offering bone-protective effects in SONFH. Future research is needed to further investigate the specific role of PRKCA in SONFH and determine whether it serves as a direct target for SZW’s therapeutic effects, providing additional evidence for SZW in treating SONFH.

According to the GSEA results, IKBKB is significantly enriched in the oxidative phosphorylation pathway, while PRKCA is enriched in the RNA degradation and ubiquitin-mediated proteolysis pathways. Both IKBKB and PRKCA may be closely associated with SONFH through multiple pathways, including the ribosome, spliceosome, proteasome, olfactory transduction, and neuroactive ligand-receptor interactions. The spliceosome, an RNA-protein complex responsible for mRNA splicing, enables alternative splicing, allowing a single gene to produce several proteins through various exon arrangements (48). Circular RNAs (circRNAs), byproducts of precursor mRNAs, act as sponges to regulate miRNA activities, facilitate gene translation *via* alternative splicing, and interact with RNA-binding proteins (RBPs) (49). Additionally, circRNAs play a role in the physiological and pathological processes of various orthopedic diseases, including osteosarcoma, osteoporosis, and osteoarthritis (50–52). Notably, our findings suggest that spliceosomes may also play a pivotal role in SONFH.

Analysis of peripheral blood immune cell composition revealed significant alterations in the proportions and activity of multiple immune cell subsets in patients with SONFH, including macrophages, neutrophils, activated CD8^+^ T cells, and central memory CD4^+^ T cells. Correlation analysis indicated that activated CD8^+^ T cells exhibited the strongest association with biomarkers, suggesting they may exert a considerable influence on the biomarkers associated with SONFH. Research has demonstrated the presence of neutrophil extracellular traps in the microvascular system surrounding the femoral head in patients with osteonecrosis, suggesting a role for neutrophils in the pathogenesis of SONFH (35). Additionally, studies have shown that macrophages and monocytes serve as sources of osteoclasts, with osteoclast-mediated bone resorption being a critical mechanism in femoral head collapse (53, 54). Previous reports have linked macrophages to bisphosphonate-related osteonecrosis of the jaw and steroid-induced osteonecrosis (55), indicating a close relationship between macrophages and SONFH. A retrospective clinical controlled study revealed that the total lymphocyte count, CD3T cells, Ts cells (CD3CD8), and B-1 cells (CD5CD19) in the peripheral blood of patients with femoral head necrosis were significantly elevated, suggesting an association between the occurrence and progression of femoral head necrosis and immune system imbalances (56). However, this study is based on blood sample analysis, directly reflecting changes in circulating immunity. The involvement of these systemic immune characteristics in the local osteonecrotic process of SONFH requires future validation using lesioned tissue samples or experimental models that more closely replicate the pathological process.

To construct TF-mRNA pairs, two TFs, MLLT1 and BCL11B, were identified as being associated with the key genes IKBKB and PRKCA. Three miRNAs associated with IKBKB, including hsa-miR-503-5P and hsa-miR-196a-5P, were identified, along with twenty-two miRNAs related to PRKCA, such as hsa-miR-24-3P. Furthermore, 260 lncRNAs regulating IKBKB and PRKCA expression were identified, with nine of these lncRNAs serving as effective predictors. In recent years, an increasing number of miRNAs have been implicated in osteonecrosis, with mechanisms such as coagulation disorders, abnormal apoptosis, and lipid metabolism dysregulation identified as contributors (57–59). Additionally, lncRNAs can competitively bind to miRNAs through the “sponge effect,” regulating the proliferation and function of osteoblasts (60). Therefore, these TFs, miRNAs, and lncRNAs may play a significant role in SONFH progression by modulating the expression of biomarkers and influencing disease progression.

This study has several limitations. First, the clinical sample size used for RT-qPCR validation was relatively small, which, although providing preliminary evidence for differential expression, may impact statistical power and limit the generalizability of the findings. Second, the validation dataset (GSE74089) used for trend observation differed from the primary training set in terms of tissue origin and disease scope, potentially introducing contextual interference in the interpretation of gene expression consistency. Additionally, due to research timeline constraints and challenges in sample collection, this study did not establish an intervention group for SZW. As a result, while upregulation of IKBKB and PRKCA was observed in patients with SONFH, it cannot be conclusively confirmed that these factors are the direct therapeutic targets of SZW. Consequently, the related conclusions remain speculative and association-based. Furthermore, although the nomogram diagnostic model demonstrated good discriminatory performance in internal data, the sample size in the training cohort was limited, and independent validation in large, homogeneous clinical cohorts was lacking. The absence of systematic comparisons with existing diagnostic methods further complicates its potential clinical applicability. Therefore, further verification is needed to assess its clinical translation.

To address these limitations, future research will focus on several directions: constructing or utilizing independent SONFH cohorts with well-matched tissues and comprehensive clinical data to validate key biomarkers; designing SZW intervention experiments to determine whether IKBKB and PRKCA serve as response targets of the formula in cellular or animal models; elucidating the specific roles of these two genes in SONFH pathogenesis and progression through gain- and loss-of-function experiments at the mechanistic level; and conducting rigorous external validation of the nomogram model, including comparative evaluations against conventional clinical diagnostic methods, to facilitate its clinical implementation. These follow-up studies are expected to advance the current correlation-based findings into causal mechanistic insights, thereby providing a stronger scientific foundation for the precise diagnosis and targeted therapy of SONFH.

## 5 Conclusion

This study leveraged public databases to download SONFH-related transcriptomic data, identifying 1,671 DEGs between the SONFH and control groups. Through network pharmacology, 69 therapeutic targets of SZW for SONFH were identified. The intersection of these 69 targets with the DEGs yielded 11 candidate genes. Machine learning, ROC curve analysis, and expression level validation were subsequently applied to identify two biomarkers (IKBKB and PRKCA) that indicate SZW’s therapeutic effects on SONFH. Finally, the potential molecular mechanisms of these biomarkers as therapeutic targets for SONFH were explored through nomogram construction, GSEA enrichment analysis, immune infiltration analysis, molecular regulatory network analysis, and molecular docking. These findings provide a new theoretical framework for understanding the mechanism of SZW in treating SONFH. However, the effectiveness of these biomarkers in diagnosing and predicting SONFH requires further validation through large-sample clinical trials. Moreover, the regulatory roles of these biomarkers in SONFH need to be experimentally validated *in vivo*.

## Data Availability

The data presented in this study have been deposited in the Gene Expression Omnibus (GEO) database, with the accession numbers GSE123568 (available at https://www.ncbi.nlm.nih.gov/geo/query/acc.cgi?acc=GSE123568) and GSE74089 (available at https://www.ncbi.nlm.nih.gov/geo/query/acc.cgi?acc=GSE74089).

## Footnotes

1
https://www.ncbi.nlm.nih.gov/geo/

2
https://old.tcmsp-e.com/tcmsp.php

3
http://herb.ac.cn/

4
https://www.uniprot.org/

5
https://www.genecards.org/

6
https://string-db.org/

7
https://github.com/psolymos/ResourceSelection

8
https://www.networkanalyst.ca/

9
http://mirwalk.umm.uni-heidelberg.de/

10
https://mirtarbase.cuhk.edu.cn/

11
https://www.mirnet.ca/

12
https://www.rcsb.org/

13
https://pubchem.ncbi.nlm.nih.gov/

14
https://cadd.labshare.cn/cb-dock/php/blinddock.php

## Glossary

SONFH: Steroid-induced osteonecrosis of the femoral head
SZW: Shenggu Zaizao Wan
DEGs: Differentially expressed genes
GSEA: Gene set enrichment analysis
RT-qPCR: Real-time quantitative reverse transcription PCR
TCM: Traditional Chinese medicine
GEO: Gene expression omnibus
TCMSP: Traditional Chinese medicine systems pharmacology database
HERB: Herbal encyclopedia for research on bioactivity
TsI: Tanshinone I
KEGG: Kyoto encyclopedia of genes and genomes
PPI: Protein–protein interaction
GO: Gene ontology
BPs: Biological processes
CCs: Cellular components
MFs: Molecular functions
LASSO: Least absolute shrinkage and selection operator
SVM-RFE: Support vector machine recursive feature elimination
ROC: Receiver operating characteristic
FDR: False discovery rate
IKBKB: Inhibitor of nuclear factor kappa b kinase subunit beta
PRKCA: Protein kinase C alpha
NIK: Nf-κb-inducing kinase
LPS: Lipopolysaccharide
circRNAs: Circular RNAs
miRNAs: MicroRNAs
RBPs: RNA-binding proteins
OB: Oral bioavailability
DL: Drug likeness
FC: Foldchange
STRING: Search tool for the retrieval of interacting genes and proteins
HL: Hosmer-Lemeshow
AUC: Area under curve
DICs: Differential immune cells
TFs: Transcription factors
cDNA: Complementary DNA
