# Supplementary figures and images for: Identification and validation of biomarkers of Shenggu Zaizao Wan in the treatment of steroid-induced osteonecrosis of the femoral head by integrating network pharmacology and bulk transcriptomic
Source: Front Med (Lausanne). 2026 Feb 4;13:1732825. doi: 10.3389/fmed.2026.1732825 (PMC12913513; doi:10.3389/fmed.2026.1732825)

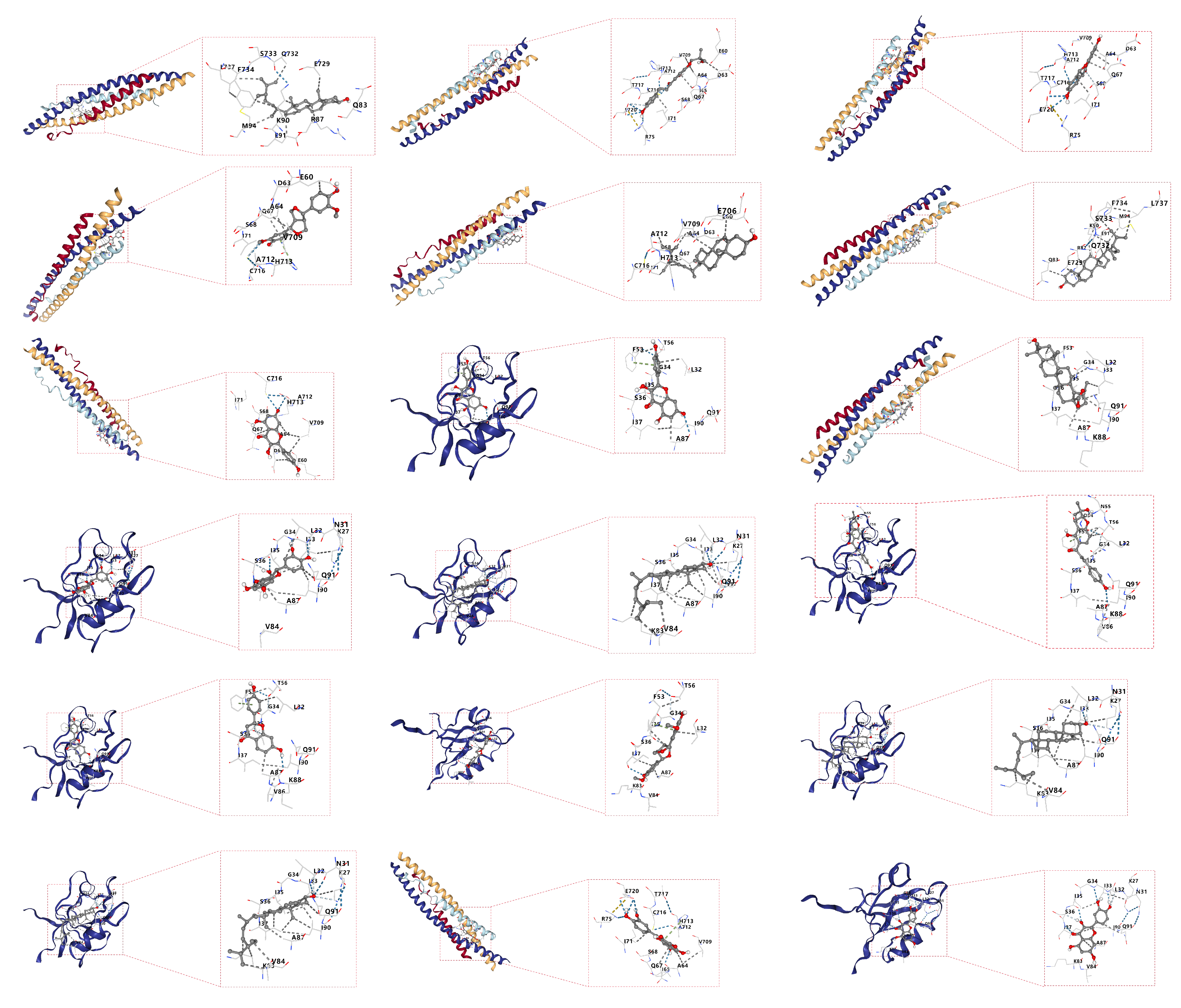

Supplement: Supplementary file 10 [file Image_1.tif]
